# Identification of New Ocellatin Antimicrobial Peptides by cDNA Precursor Cloning in the Frame of This Family of Intriguing Peptides

**DOI:** 10.3390/antibiotics9110751

**Published:** 2020-10-29

**Authors:** Mariela M. Marani, Silvana Aguilar, Ana P. Cuzziol Boccioni, Natalia L. Cancelarich, Néstor G. Basso, Fernando Albericio

**Affiliations:** 1 IPEEC-CONICET, Consejo Nacional de Investigaciones Científicas y Técnicas, Bvd. Brown 2915, U9120ACD Puerto Madryn, Argentina; saguilar@cenpat-conicet.gob.ar (S.A.); anapaulacuzziolboccioni@gmail.com (A.P.C.B.); ncancelarich@cenpat-conicet.gob.ar (N.L.C.); 2 FBCB-UNL, Facultad de Bioquímica y Ciencias Biológicas, Universidad Nacional del Litoral, Casilla de Correo 242, S3000 Santa Fe, Argentina; 3 IDEAus-CONICET, Consejo Nacional de Investigaciones Científicas y Técnicas, Bvd. Brown 2915, U9120ACD Puerto Madryn, Argentina; nbasso@cenpat-conicet.gob.ar; 4 Peptide Science Laboratory, School of Chemistry and Physics, University of KwaZulu-Natal, 4001 Durban, South Africa; albericio@ukzn.ac.za; 5 Institute for Advanced Chemistry of Catalonia (IQAC-CSIC), 08034 Barcelona, Spain; 6 CIBER-BBN, Networking Centre on Bioengineering, Biomaterials and Nanomedicine, and Department of Organic Chemistry, University of Barcelona, 08028 Barcelona, Spain

**Keywords:** in silico techniques, leptodactylidae, *Leptodactylus latrans*, natural products, bioprospection

## Abstract

Ocellatins are a family of antimicrobial peptides found exclusively in the *Leptodactylus* genus. To date, 10 species have been studied and more than 23 peptides described. Here we report the sequences of five new peptides from the skin of the frog *Leptodactylus latrans* (Anura: Leptodactylidae) determined by cDNA cloning of the complete prepro-peptide structures. The mature peptides were characterized with in silico tools and compared with those previously described. With 21 amino acid residues, this new set of peptides not previously described in the *Leptodactylus* genus share between 100 and 76.2% similarity to ocellatin antimicrobial peptides. These novel peptides are cationic and their three-dimensional (3D) structure holds the highly conserved residues G^1^, D^4^, K^7^, and K^11^ and a high theoretical amphipathic α-helix content. Furthermore, in silico analyses of these new peptides predicted antimicrobial activity. This study is framed in the context of previous work published about ocellatins, and therefore, provides a review of this intriguing family of peptides.

## 1. Introduction

Antimicrobial peptides (AMPs) are short polypeptides found in the species of six kingdoms and they play a crucial role in innate immune systems. In amphibians, skin secretions have at least two main functions: host-shielding antimicrobial activity and a toxic effect against predators [1,2]; therefore, research into amphibian AMPs is focused mainly on the design of new drugs to treat human diseases. There is increasing concern about the antibiotic resistance of several pathogenic microorganisms, and thus, an urgent need to tackle this problem [3,4]. Despite drawbacks [5,6], AMPs are a novel class of molecules with a broad spectrum of activity and low capacity to induce bacterial resistance [7]. In combination with advanced chemoinformatics tools, they offer the possibility to address obstacles by peptide design approaches [8,9].

Despite advances in molecular and cell biology, in silico design, and automatization such as high-throughput screening, natural products are still one of the most successful sources of drug leads. The impact of natural products on drug discovery is not limited to the peptide domain but extends to all types of drugs [10]. Although recent years have witnessed revived interest of the pharmaceutical industry in natural products, many analysts attributed the crisis that this industry experienced at the end of the last century to the closure of most internal Natural Product departments, which had been in place since the 1980s.

In the field of peptides and natural products there are several interesting examples to highlight as Ziconotide (Prialt^®^), a potent analgesic discovered from the venom of a marine cone snail of the *Conus magus* species, and Romidepsin (Istodax^®^), first isolated from the bacterium *Chromobacterium violaceum* found in a soil sample in Japan that has a potent anticancer activity against some non-Hodgkin lymphomas.

As a naturally occurring antibiotic peptide, we can mention Daptomycin, isolated from the actinobacterium *Streptomyces roseosporus* in the soil and used in hospitals for the treatment of serious infections caused by multi-drug resistant bacteria and Colistin (or Polymyxin E), both cyclic peptides. Colistin was first isolated in Japan during the fermentation of *Bacillus polymyxa var*, and it is also used for infections that are difficult to treat. A good example of a peptide arising from frog skin is Pexiganan, a synthetic analog of magainin 2 derived from *Xenopus laevis* skin that reached phase III clinical trials as a topical antimicrobial agent for diabetic foot ulcers. The history of this peptide reflects the long and risk-laden process of drug discovery. Magainin 2 was identified in 1987. In 1999, the Food and Drug Administration (FDA) rejected the application of Magainin Pharmaceuticals, but another company, Dipexium Pharmaceuticals, ran the drug until phase III clinical trials but in 2016 failed to achieve FDA authorization again. This is not a problem unique to peptides as drugs but rather a dominant issue in the pharmaceutical industry, where approximately 1 out of every 10,000 or more compounds identified and analyzed reach the market [11]. However, peptides as potential drugs are considered to be “friendlier” than small molecules in this drug discovery journey due to the ease of analog synthesis and lack of toxicity, which, therefore, facilitates the progress of these biomolecules through the different phases and makes them a viable alternative to small molecules. 

In the field of AMPs, more than 3240 natural AMPs enrich databases [12]. With computer-assisted design and bioinformatics analysis strategies, researchers can anticipate and optimize peptide properties and launch medicinal chemistry programs. Such programs involve data set preparation, peptide representation, model construction, design, and optimization of the active AMP.

AMPs are highly diverse. They can be categorized into different families on the basis of their structure and physical-chemical properties. Magainins, caerins, dermaseptins, brevinins, esculentins, and ocellatins are some of the types that have been described in amphibians [13,14]. The first ocellatin peptides were reported in the species *Leptodactylus ocellatus* (now *L. latrans)* [15,16], to which they owe their name. Members of the ocellatin family were described only in the skin secretion of frog species belonging to *Leptodactylus*. Grouping 78 species, *Leptodactylus* is a widely distributed genus throughout the south of North America, South America, and the West Indies [17]. Ocellatins have been identified by direct de novo sequencing using Edman′s mass spectrometry and microsequencing techniques, although molecular biology techniques to isolate mRNA and clone the full cDNA of complete prepro-peptides have also been performed [18].

Using molecular biology techniques, the present study aimed to identify novel complete prepro-peptide structures of AMPs in the skin of a specimen of *L. latrans* collected in Chaco, Argentina. The newly identified mature peptides were studied in relation to the ocellatin peptide family, comparing their physical-chemical characteristics and analyzing their sequences and theoretical structures to predict their activity against pathogen strains. 

## 2. Results and Discussion

### 2.1. Identification of Peptide-Encoding cDNA Sequences 

**Figure 1:**
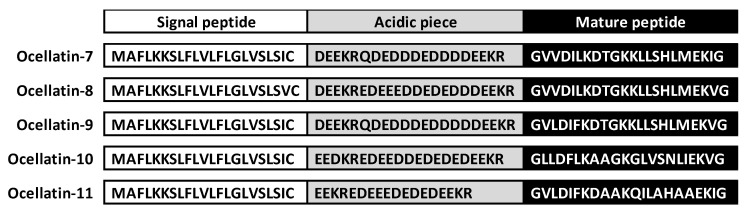
Deduced amino acid sequences of the ocellatin precursors of *Leptodactylus latrans*. Prepro-regions (signal peptide and acidic region boxed white and gray respectively) and a variable domain (boxed black) that correspond to mature peptide are indicated. Cys residue indicates the end of the signal peptide and the Lys–Arg processing site shows the end of the acidic region.

To identify the sequences of new peptides from the skin of the frog *L. latrans,* total RNA was isolated, followed by amplification and sequencing of specific cDNAs of the prepro-peptide precursors. After cloning the cDNA structures, 83 colonies were selected and grown separately in liquid media for subsequent plasmid purification. Size evaluation of the insert fragments was performed for selection and posterior sequencing (Figures S1 and S2). The nucleic acid and the deduced amino acid sequences of cDNA encoding new ocellatins are detailed in the supporting information (Figure S3). The results, as shown in Figure 1, indicate the identification of five de novo sequences of complete prepro-peptides with a typical AMP tripartite structure, namely, a 21-residue long highly conserved signal peptide ending with the characteristic Cys residue; variable acidic domain (19–22 residues), which present the typical Lys-Arg prohormone processing signal at their carboxyl terminus; and the mature peptide. All mature peptides presented a Gly residue at the C-terminal end of their sequences, which involved the amidation of the peptide at that end [19].

### 2.2. Structural Comparison and Nomenclature

Comparison of mature peptide sequences with the peptides deposited in the APD and DRAMP databases confirmed the identification of a set of five previously unknown peptides with high degrees of similarity between them and to AMPs of the ocellatin family [20,21]. Since *L. latrans* was defined originally as *L. ocellatus* [22], from which the name of ocellatin-1 to -6 was derived, we did not incorporate letters and the newly identified peptides were named ocellatin-7 to -11, following the proposed nomenclature [23,24,25].

A comparison of the primary structures of the novel peptides showed highly conserved residues. Six amino acid residues (Gly^1^, Asp^4^, Lys^7^, Lys^11^, Glu^19^, and Lys^20^) are invariant, and 11 residues present conservative substitutions (Figure S4). Pairwise alignment between the identified peptides using DRAMP revealed between 52.4 and 100% similarity (Table S2), the highest values being between ocellatin-7 and ocellatin-9 and ocellatin-8, which show 90% and 100% of similarity (90.5% and 95.2% identity) to each other, respectively. 

As shown in Table 1, ocellatin-11 presents 95.2% of similarity to ocellatin-2. Ocellatin-9, -8, and -7 show 76.2% similarity to ocellatin-5. Ocellatin-10 is 86.4% similar to ocellatin-6 and interestingly presents 90.47% identity and 95.5% of similarity to the peptide analog P3-Ll-2085, a hybrid peptide combination of two fragments [26].

### 2.3. The Ocellatin Family

The information described in this section was obtained after an exhaustive search of the Antimicrobial Peptide Database (APD3), the Database of Antimicrobial Activity and Structure of Peptides (DBAASP), DRAMP, and BLAST and an in-depth analysis of the literature. In all cases, the isolation and identification method, specific associated activity, structural analysis, and study of the mechanism of action were considered (Table S1).

To date, a set of 28 ocellatin peptide sequences, including the five reported in the present study, have been described in 10 species of the genus *Leptodactylus* (Table 2). Ocellatin-1 to -3 [15], ocellatin-4 [16], ocellatin-5 and -6 [27], and ocellatin-7 to -11 (this work) were identified in the skin secretion of *L. latrans* (formerly *L. ocellatus*). Ocellatin-F (fallaxin) was identified in *L. fallax* [28]; ocellatin-P (pentadactylin) in *L. pentadactylus* [29]; ocellatin-L1 (laticeptin) and ocellatin-L2 in *L. laticeps* [30]; ocellatin-S (syphaxin) in *L. syphax* [31]; ocellatin-V1 to -V3 in *L. validus* [32]; ocellatin-K1 in *L. knudseni* [33]; ocellatin-PT1 to -PT8 in *L. pustullatus* [18]; and ocellatin-LB1 and -LB2 in *L. labyrinticus* [34]. In our opinion, these last two peptides should have been classified as ocellatin-F (1–22) and ocellatin-F (1–23) as their sequences are fragments of ocellatin-F (see below). It is interesting to note that ocellatin-F and ocellatin-K1 were identified in more than one species of the *Leptodactylus* genus. Ocellatin-K1 was present not only in *L. knudseni* but also in *L. vastus* [35], whereas ocellatin-F was present in three species, namely *L. fallax*, *L. pentadactylus*, and *L. labyrinticus* [29,34].

As observed in the literature, numerous ocellatin fragments were also identified by de novo sequencing techniques in the skin secretions of leptodactylid frogs (Table S3). Ocellatin-S (1–16), (1–22), (1–23), (1–24), and (16–25) were present in the skin secretion of *L. syphax*; ocellatin-K1 (1–16), and (1–21) in *L. vastus*; and P2-Ll-1298 in *L. latrans* [26]. Ocellatin-F (1–22) was present in the skin secretion of *L. fallax* [28] and ocellatin-F (1–23), named Des-Lys24-Leu25-ocellatin-F1, was present in the skin secretion of *L. labyrinticus.* Two ocellatin-F fragments, ocellatin-LB1 and -LB2, were also identified in *L. labyrinticus* [34]. In our opinion, these peptides should be described as ocellatin-F (1–22) and (1–23), since their sequences do not present originality with respect to the peptides already described, and even ocellatin-F was present in the secretion of that species. The fragment ocellatin-L1 (1–22) was found in *L. laticeps.* Five fragments were identified in skin secretion of specimens of *L. latrans* (ocellatin-1 (1–16), ocellatin-2 (1–15), ocellatin-3 (1–15), ocellatin-5 (1–14), and ocellatin-6 (1–18)) [27]. Furthermore, Nascimento et al. described ocellatin-5 [36]. Given its length (17 amino acid residues) and the presence of ocellatin-5 (1-16), we propose that this ocellatin-5 is indeed a fragment of a distinct undescribed ocellatin. Considering that Leite Jr. et al. [27] have been described a different 21-residue sequence as ocellatin-5, we propose to call the first one ocellatin-5* until definitive identification of the corresponding ocellatin. The ocellatin-5* sequence is almost identical to the N-terminal region of ocellatin-10, except for one residue (T15S). Table S3 summarizes the experimental data obtained to date for the antimicrobial activity of ocellatin fragments. 

Interestingly, the skin secretions of leptodactylid frogs also contain peptides with remarkably different sequences, such as LASP (GLWDDLKAAAKKVVSSLASAAIEKL-NH_2_), which have been described as *Leptodactylus* aggression-stimulating peptides in male frogs of *L. fallax* [37]. Gly-rich peptides have also been described: plasticin-L1 (GLVNGLLSSVLGGGQGGGGLLGGIL-OH) in *L. laticeps* [38], and leptoglycin (GLLGGLLGPLLGGGGGGGGGLL-OH) in *L. pentadactylus* [39]. Curiously, anionic peptide P1-Ll-15577 (DEMKLDGFNMHLE-NH_2_) was also reported in *L. latrans* [26]. These types of peptide are unusual in frog secretions, although several have been identified in other species [40,41]. 

Numerous synthetic peptide analogs have also been designed from ocellatins to improve activity. The peptide KLLKFVTKVGKAIFKALIKAI-OH is an ocellatin-4 analog. It has an increase in the positive net charge from +1 to +6, which improves its activity against plant pathogens [42]. P3-Ll-2085 [26], a hybrid peptide combination of two fragments (the N-terminal region of ocellatin-5* and the fragment P2-Ll-1298, identified in the secretion of *L. latrans*) showed a minimal inhibitory concentration (MIC) of 15 µM. Furthermore, analogs of ocellatin-F and ocellatin-4 were also designed for structure-activity relationship studies (SAR) [43].

### 2.4. Amino Acid Frequency and Particular Motif Occurrence

The peptides of the ocellatin family described hitherto contain 21, 25, or 32 residues, are cationic, and present conserved positions. However, they usually present a low cationic character, varying between net charges +3 and +1, compared with other peptides isolated from the skin of amphibians, which have double or triple loads, such as esculentin-2P from *Rana pipiens* with a net charge of +6, hymenochirin-1Pa from *Pseudhymenochirus merlini* with +7, or cathelicidin-RC1 and -RC2 from *Rana catesbeiana* with +9 and +8, respectively [44,45,46]. The positive character depends not only on the balance between positive and negative charges but also on the posttranslational amidation of the C-terminus, all ocellatins being amidated at the C-terminus, with the exception of the longest ones.

**Figure 2:**
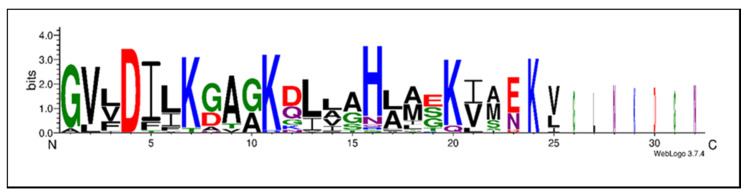
Sequence motifs common to the 28 ocellatin family peptides described to date. The amino acids are stacked on top of each other in increasing order of frequency and plotted. The height of each letter is proportional to the observed frequency of the corresponding amino acid. The overall height of each stack is proportional to the sequence conservation at that position. Amino acid color code: green = polar (G, S, T, Y, C, Q, N), blue = basic (K, R, H), red = acidic (D, E), and black = hydrophobic (A, V, L, I, P, W, F, M).

Only one residue, namely Asp_4_, is invariant for all the ocellatins described. However, several positions are highly conserved in the 28 ocellatins (Table 2). The distribution of acidic and basic amino acids plays a central role in the approach to the bacterial membrane. Analysis of the ocellatin sequences identified preferred locations for the basic and acidic residues. Sites 7, 11, 20, 24, and 29 are practically exclusive for the Lys residue and site 16 for a highly conserved His (Figure 2). As expected, SAR studies showed that Lys seems to have a greater effect than His because replacing the positively charged Lys in sites 7, 11, 20, and 24 with Ala results in a loss of activity compared to the original peptide, while the substitution of the His_16_ residue with Ala_16_ has no observable effect on antibacterial or hemolytic activity [43]. 

Acidic amino acids also have preferred sites. In general, ocellatins have no more than two or three acidic amino acids, except for ocellatin-PT6. Site 4 of ocellatins is exclusively for the residue Asp. However, the second or third acidic residue is located at sites 8, 12, 19, or 23, positions 8 or 12 being excluding sites. At sites 19 and 23, the residue Glu is present instead of Asp, site 19 being preferred in short ocellatins. In the case of longer ocellatins (32 residues), described to date only in *L. pustullatus*, an acidic Asp residue is present at the C-terminal end at position 30. However, these particularly long ocellatins do not have acidic amino acids at site 8 or 12. 

It is important to note that ocellatins maintain a low cationic character through a combination of positive and negative residues since they conserve both the basic amino acids and the number of acid residues in specific positions. Acidic and basic residues play a crucial role in the electrostatic attraction between charged peptides and negatively charged bacterial membranes. Therefore, higher positively charged peptides will have a greater capacity to interfere with the membranes of microorganisms. SAR studies with ocellatins demonstrated that the replacement of acid residues by Lys or by the electrically neutral Ala leads to an increase in antibacterial activity [36,43]. However, to date, no ocellatin without acidic residues has been described, and Asp_4_ is the only residue exclusively preserved in all ocellatins. 

The five new ocellatins identified herein belong to the group of shortest ocellatins. They present 21 residues and a +2 net charge for ocellatin-7, -8, -9, and -10 and +1 for ocellatin-11. All of these compounds have Asp_4_, Lys_7_, Lys_11_, Glu_19_, and Lys_20_, whereas Asp_8_ and His_16_ are present in four of the five peptides. Translation of the nucleotide sequences of the prepro-peptides revealed the presence of a Gly residue at their C-terminal end. This residue represents a signal for C-terminus amidation during the posttranslational maduration process of the peptides. C-terminus amidation is common in natural AMPs, where a difference of approximately 1u is observed between the theoretical and experimental molecular masses according to the amino acid sequence [18,47]. All ocellatins are amidated at the C-terminus. Only the longer ocellatins present an acidic C-terminus (ocellatin-PT6, -PT7 and -PT8). C-terminus amidation is essential for the biological activity of many neuropeptides and hormones [48]. Several studies have proposed that C-terminus amidation confers enhanced antimicrobial activity compared with analogs with an acidic C-terminus, although this effect is not universal [49,50].

**Figure 3:**
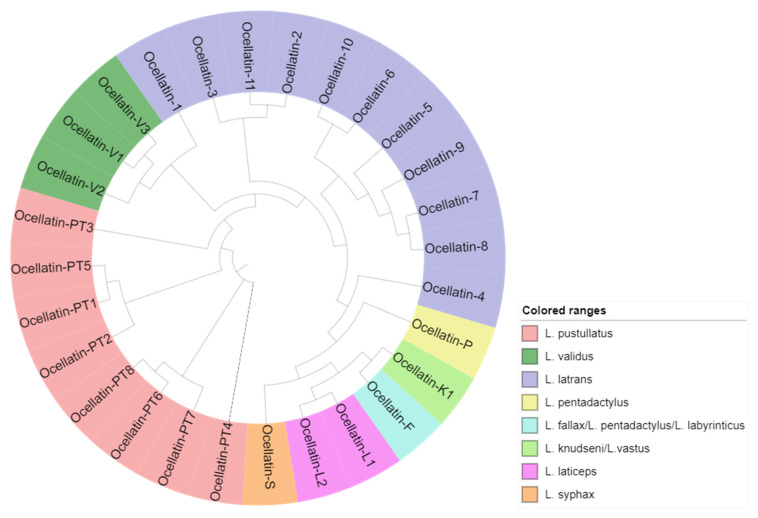
Cladogram of all ocellatin peptides identified from the *Leptodactylus* genus to display similarity, and the associated anuran species in which they were identified (following the color code).

Hydrophobic amino acids also play a significant role in antimicrobial activity performance. Most natural peptides have a 40–60% content of hydrophobic residues, which are generally located in a specific sector on a helical wheel projection, thereby giving an amphipathic character to the molecule [51]. Of note, all ocellatins have hydrophobic residues at fixed positions among the charged residues, these being conservative substitutions. This arrangement results in the motif GXXDXXK for most ocellatin peptides, where X could be hydrophobic amino acids such as Val, Leu, Ile, and Phe, with a preference for Val in position X_2_ and Ile in position X_6_. These aliphatic amino acids are also typical in other positions such as X_13_, X_14_, X_17_, X_21_, and X_25_ (Figure 2 and Figure 3). These arrangements seem to be related to the phylogeny between *Leptodactylus* species, where ocellatin peptides identified in *L. syphax, L. laticeps, L. vastus, L. knudseni,* and *L. fallax* are phylogenetically close, differing only in conservative residues. However, certain positions present radical substitutions or replacements. Site 8 varies between small amino acids, such as Gly in *L. vastus,* and the electrically charged Asp in *L. latrans* and *L. pustullatus*. Position 12 varies between simple residues such as Gly or amino acids with acidic (Asp), basic (Lys), or polar uncharged (Gln, Asn) side chains. While in position 19, Glu is present for most of the ocellatins isolated from *L. latrans,* in *L. vastus* this position is occupied by Ser and in *L. pustullatus* by Gly. Some hydrophobic residues play a crucial role in the activity of ocellatins. In this regard, the replacement of Leu with Ala results in decreased activity compared with the original peptide [43].

### 2.5. Phylogenetic Relationships

Analysis of the alignment of the ocellatin peptides (Table 2) and its associated cladogram (Figure 3) reveals that although the pressure to conserve the complete amino acid sequences of the peptides has been weak, with only one invariable residue (Asp_4_), there is a structure relationship indicative of a common evolutionary origin. De Sá et al. analyzed more than 80% of the recognized species of *Leptodactylus* and, based on a combined analysis of molecular and non-molecular data, suggested that evolution in this genus took the form of a pectinate tree with four main groups. A basal position group called *L. fuscus* (36 species), followed by the *L. pentadactylus* species group (17 species), which is the sister group to *L. latrans* (10 species), and the *L. melanonotus* (17 species) group [52].

Among the 10 species of *Leptodactylus* from which ocellatin peptides were identified, there are species of the four proposed groups. Two belong to the *L. fuscus* group (*L. syphax* and *L. laticeps*), five to the *L. pentadactylus* group (*L. vastus, L. fallax, L. labyrintycus, L. knudseni,* and *L. pentadactylus*), one to the *L. latrans* group (*L. latrans*), and two to the *L. melanonotus* group (*L. pustullatus* and *L. validus*). 

It is noteworthy that the peptides from *L. vastus, L. fallax, L. labyrinticus, L. knudseni,* and *L. pentadactylus* are grouped, thereby validating the common origin perceived in the phylogram, and the same was observed for *L. pustullatus* and *L. validus*. All the new peptides identified from *L. latrans* in this work are segregated, together with four of the six ocellatins previously described in specimens of the same species. Ocellatin-10 and -6 form one cluster; ocellatin-9, -7, -8, and -5 form another sister cluster, and ocellatin-11, -2, and -3 are in a third cluster. 

Some authors [13,53] have postulated that skin peptide profiling can be employed in combination with other sources of information, such as molecular and morphological characters, to discriminate hybrids, species, and clades at higher taxonomic levels. However, a systematic analysis to evaluate this approach has not been conducted to date. Furthermore, since peptide profiles can fluctuate depending on environmental factors [54], an extensive sampling and deeper understanding of the variability and evolution of these substances are required before considering their use in systematic [2].

### 2.6. Physical-Chemical Properties, 3D Structure, and Activity Prediction 

To predict the activity of the novel ocellatins, we carried out a comparative study of the physicochemical parameters of the peptides identified with those already described.

The activities of AMPs and their ability to disrupt the plasma membrane of eukaryotic cells are dependent upon complex interactions between charge, hydrophobicity, conformation (helix stability), and amphiphathicity [55,56]. 

#### 2.6.1. Physical-Chemical Properties

**Figure 4:**
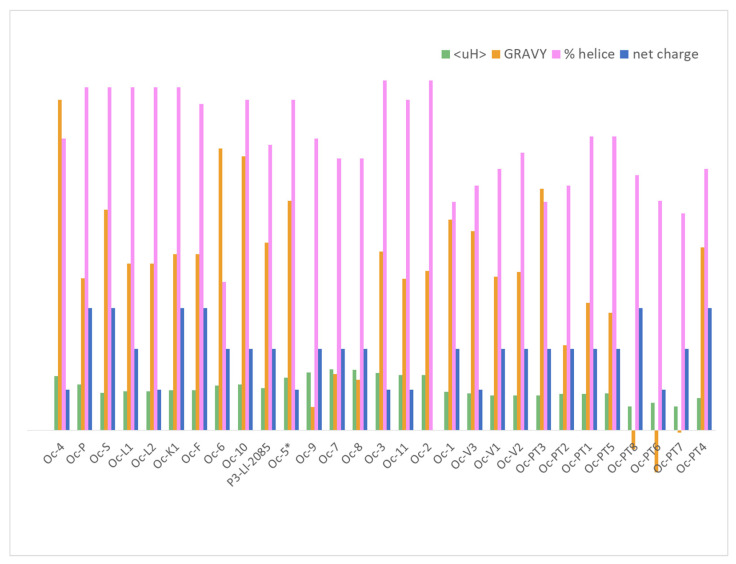
Physical-chemical properties of ocellatins. Net charge, hydrophobicity (GRAVY), amphipathicity (µH), and percentage of α-helix content.

All the ocellatins described to date are cationic peptides and they present a high α-helix content prediction, and therefore, high values of pI, between 9.66 and 10.7, except ocellatin-2, which is the only member of this family with a net charge of 0 (Zero). Three-dimensional (3D) structure analysis using NMR and CD experiments has been performed for only a few ocellatins. NMR of ocellatin-F1 showed well defined helical segments along the polypeptide 3D structures. In the presence of micelles, ocellatin-F1 is bent and the helix curvature is consistent with the internalization of the hydrophobic residues into the membrane interior [57]. Additionally, although in aqueous media most of tested peptides present spectra consistent with random coil conformations, CD techniques demonstrated an enhancement of the helical contents with high percentages of α- helix in the presence of trifluoroethanol (TFE), dodecylphosphocholine (DPC), sodium dodecyl sulfate (SDS), or vesicles with dipalmitoyl phosphatidylglycerol (DPPG), 1-palmitoyl-2-oleoyl-sn-glycero-3-phosphocholine (POPC), and POPC:1-palmitoyl-2-oleoyl-sn-glycero-3-phospho-L-serine (POPG) [3,26,34]. These observations indicate that the hydrophobic environment and the negative charge density of the membranes induce the adoption of such a structure of the peptide, and therefore, subsequent interaction. Ocellatins-LB1, -LB2, and -F1 from the skin of *L. labyrinticus* acquire higher helical contents, reaching approximately 90% in the presence of phospholipid vesicles, when compared to the peptides in the presence of TFE-H_2_O or aqueous micellar solutions [34]. The predicted α-helix content for the rest of the ocellatins is between 53–86%, except ocellatin-6 (Figure 4 and Figure S5C).

To interact with negative density charged bacterial membranes not only must the peptide have a positive net charge but also a high hydrophobic content, at least 50%, and an amphipathic arrangement. The GRAVY value is a useful index to evaluate hydrophobicity. It is calculated by adding the hydropathy values [58] of each amino acid residue and dividing by the number of residues in the sequence. A higher positive score indicates increased hydrophobicity. Figure 4 shows the extensive variability of hydrophobicity among ocellatins, which GRAVY values range between −0.103 and 0.81, the aggregation value also being variable (Figure S5B,C). The ocellaltin-10 described by our group is among the most hydrophobic ocellatins (together with ocellatin-4, -5, and -6), while the novel ocellatin-7, -8, and -9 are the least hydrophobic. Ocellatin-PT6, -PT7, and -PT8 are the only hydrophilic peptides in the ocellatin family (Figure 4).

#### 2.6.2. D Theoretical Structures

**Figure 5:**
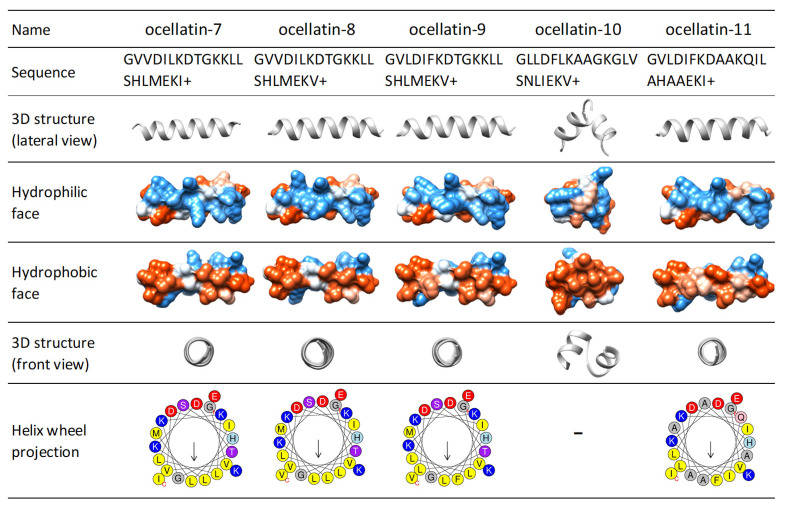
Three-dimensional (3D) structure prediction, hydrophobic, and hydrophilic faces and Schiffer and Edmundson helical wheel projection diagrams of the peptides identified from the skin secretion of *Leptodactylus latrans*. Diagrams show α-helix motifs with amphiphilic peptide structures having a hydrophobic (red region) and hydrophilic region (blue region). Amino acid color code for the wheel projections: yellow = non-polar/hydrophobic (Leu, Val), gray = Gly, blue = basic (Lys, Arg), purple = polar without charge (Thr), pink = polar without charge (Asn), and green = Pro.

The 3D theoretical structures of ocellatins predicted by the PEP-FOLD server vary between two types: a single α-helix (15 ocellatins, Figure S6) or an α-helix linked by a kink (13 ocellatins, Figure S7). An analysis of the sequences revealed that the amino acid residue at position 10 and the surrounding amino acids are decisive in defining the 3D structure adopted (see supporting information, 3D structure section). Ocellatin-7, -8, -9, and -11 are predicted as a single α-helix, while ocellatin-10 shows a structure with two helices joined by a coil (Figure 5).

The arrangement plays a significant role in the balance between charged and hydrophobic amino acids. It should be amphipathic, with a hydrophilic side and an opposite hydrophobic side in the projection of the helical wheel. The amphipathic helix improves activity and selectivity, although some studies have observed that an imperfect amphipathicity provides certain benefits [59]. The hydrophobic moment is a measure of the amphipathicity of a helix. Each amino acid side chain is assigned a positive or negative value on the basis of its hydrophobicity. These values are used to weight vectors for each residue as they are displayed around the helix [60,61]. Ocellatin peptides show variable hydrophobic moments (between 0.6–1.49) (Kyte-Doolittle scale), with values that indicate amphipathic helical structures. The Schiffer and Edmundson wheel projection diagram allows visualization of the arrangement of amino acids in α-helix type structures. Figure S8 shows the wheel projections of all ocellatins that have a single-α-helix theoretical 3D. Among the single-α-helix structured ocellatins, although the newly identified ocellatin-9, -7, -8, and -11 are the least hydrophobic, they present the highest hydrophobic moments (Figure 5 and Figure S4A,B).

#### 2.6.3. Activity Prediction 

Table 3 shows the compilation of the experimental results of the antimicrobial activities of the ocellatin family peptides tested thus far against two characteristic Gram-negative and Gram-positive strains. 

Comparison of the physical-chemical parameters of all members of the ocellatin family (Figure 4) with their experimental activity (Table 3) shows that antimicrobial activity does not depend on a specific combination of these parameters but is rather more complex. The antimicrobial activity of a peptide cannot be predicted solely on the basis of physical-chemical parameters as a different combination may result in peptides with similar activity and vice versa. As shown elsewhere, the MICs of peptides with the same charge, <H>, and µH differ by 10 orders of magnitude [43]. These parameters are useful when comparing peptides derived from the same peptide. 

Ocellatins show variable antimicrobial activity against *E. coli*, with MICs ranging from 14 to 320 µM. Additionally, innovative activities were tested for ocellatin family members as combining the antiviral activity with alkaloid, antioxidant activity, or neuromodulatory therapeutic applications. Ocellatin-F1, cooperatively with bufotenine, presented an antiviral effect on the inhibition of rabies virus infection in BHK-21 cells [62]. Additionally, ocellatin-K1 fragments increased superoxide dismutase activity and glutathione concentration, as well as preventing the formation of reactive oxygen species (ROD) induced by lipopolysaccharides [35]. Moreover, within the ocellatin family, peptides with synergistic properties with antibiotics and antibiofilm activity have been identified against the multidrug-resistant strain *Pseudomonas aeruginosa* [3]. An ocellatin-4 analog has also been proposed for the control of bacterial pathogens affecting agriculture [42].

We focused on the physical-chemical properties and available experimental data on the activity of ocellatins-2, -5, and -6 since they are the ocellatins that present the highest percentages of similarity to the newly identified peptides. Ocellatin-2 is 99% similar to ocellatin-11, ocellatin-5 is 76% similar to ocellatin-7, -8, and -9, and ocellatin-6 is 86.4% similar to ocellatin-10 (Table 1). We also considered the synthetic peptide P3-Ll-2085 since it is 90.5% identical and 95.2% similar to ocellatin-10, differing in only one amino acid (I18L) (preserved substitution) (Table 1).

The MICs of ocellatin-5 against *E. coli* and *S. aureus* are 64 µg/mL (28 µM) and 128 µg/mL (56 µM), respectively [27] (Table 3). An in silico tool indicates that ocellatin-5 has a 0.81 chance of being an antimicrobial peptide. Although ocellatin-7, -8, and -9 show similarities of 71–76% to ocellatin-5, the prediction programs indicate that the probability of them being AMPs is less than 0.4 (Table S1). The main difference between these ocellatins is their hydrophobicity and 3D structure. Ocellatin-7, -8, and -9 show much lower hydrophobicity (GRAVY 0.14-0.06) than ocellatin-5 (GRAVY 0.56). They present a hydrophobic residue percentage of 42%, while ocellatin-5 has 52%. They also show differences in the % of α-helix, with lower percentages for ocellatin-7, -8, and -9, between 66 and 71%, whereas ocellatin-5 presents >80% (Table S1). Ocellatin-7, -8, and -9 show very high similarity (90.5–100%), and the residues in which they differ correspond to conservative substitutions. We expect them to behave in a similar fashion and not to exceed the antimicrobial capacity of ocellatin-5. 

Ocellatin-11 shows high similarity to ocellatin-2, differing in only one amino acid (K20Q). In preliminary antimicrobial assays, ocellatin-2 inhibited the growth of *E. coli* but at millimolar level (probably influenced by its zero-net charge). Both peptides present very similar physical-chemical characteristics (hydrophobic moment value, (µH), hydrophobicity (GRAVY), percentage of α-helix, and percentage of aggregation (Table S1 and Figure 4**)**. Therefore, considering that ocellatin-11 presents a net charge of +1, it is conceivable that it could outperform ocellatin-2 in terms of activity against these strains. The influence of the charge in the mechanism of action of AMP was widely studied [63,64]. SARS studies demonstrate that charge played a greater role in translocation efficacy of the peptides than hydrophobicity, with a higher net positive charge leading to a higher level of translocation into bacterial or *C. albicans* membranes [65]. The prediction of the penetration depth of the DBAASP tool for ocellatin-11 also supports this observation. The value of penetration depth is even slightly higher than for ocellatin-2 (23 vs. 22 for ocellatin-10 vs. ocellatin-2, Table S1). Therefore, this additional change could improve the interaction of the peptide with the membrane.

However, ocellatin-10 has a better projection since its sequence differs from P3-Ll-2085 in only one conservative substitution (I18L). P3-Ll-2085 shows activity of 15 µM against both *E. coli* and *S. aureus* (Table 3**)**. No significant differences in the values of most of the physical-chemical parameters of the two peptides were observed (Figure 4 and Table S1). Both peptides present the same net charge (+2), µH, and GRAVY. However, there is a difference in the percentage of aggregation, with ocellatin-10 presenting an intermediate value between ocellatin-6 (32%) and P3-Ll-2085 (7%). Given these observations, we do not consider this parameter relevant for activity projection. Although ocellatin-10 differs from ocellatin-6 in the percentage of α-helix (81 vs. 36%), it has a similar value to P3-Ll-2085 (70%). Circular dichroism studies of P3-Ll-2085 in different environments [H_2_O, TFE-H_2_0 (50% *v*/*v*)], and in the presence of DPPG or DPPC vesicles confirmed the adoption of a helical conformation in TFE and DPPG with over 70% of α-helix content. The theoretical 3D structure prediction indicates that ocellatin-6, ocellatin-10, and P3-Lla-2085 have very similar arrangements, with two helices joined by a slit (Figure S7). Therefore, the similarities between the identified peptides and those already described lead us to expect that ocellatin-10 will have a MIC of around 15 µM.

## 3. Materials and Methods

### 3.1. Amphibian Collection

One adult specimen of *Leptodactylus latrans* was collected from a peri-urban site in Resistencia, Chaco, Argentina 27°23′50.9″ S 58°55′33.5″ W (−27.397460, −58.925960), (Transit Guide No. 5144, and authorization of the Subsecretary of Environment and Biosafety of the province of Chaco). The specimen was deposited in the herpetological collection, voucher numbers CNP 4208, Colección Herpetológica-Anfibios of the Instituto de Diversidad y Evolución Austral (IDEAus)—CONICET. The authors state that all animal manipulation was carried out following the ARRIVE guidelines [66].

### 3.2. cDNA Cloning

The dorsal skin of a *L. latrans* specimen (previously anesthetized with lidocaine hydrochloride) was isolated, frozen in liquid nitrogen, and pulverized in a mortar using the Trizol (Invitrogen) reagent to extract total RNA. Agarose gel was used for quality analysis of RNA. Reverse transcription and synthesis of first-strand cDNA were performed using M-MLV Reverse Transcriptase (Promega) and the primer 008 (5′- GACCACGCGTATCGATGTCGACTTTTTTTTTTTTTTTT-3). After synthesis of the first strand, the 3′RACE procedure was performed using a primer that anneals to a conserved region as a forward primer, and 002 (5′-ATGGCTTTCCTGAAGAAATCTCTTTTCCTTGTACTATTCCTTGG-3′) and 009 (5′- GACCACGCGTATCGATGTCGAC-3′) as the reverse primer. The cycling parameters were as follows: one cycle of 94 °C/120 s; four cycles of 94 °C/60 s, 53 °C/60 s, and 72 °C/60 s; 35 cycles of 94 °C/30 s, 53 °C/30 s, and 72 °C/60 s; and one cycle of 72 °C/420 s. The PCR products were purified using ADN PuriPrep-GP Kit (Highway) and linked to the pCR4-TOPO (TOPOTA, LifeTechnologies) for sequencing (Invitrogen). *Escherichia coli* Top 10 competent cells were prepared and transformed using the Inoue method described by Sambrook and Russell [67]. Following the selection and growth of bacterial colonies, the resulting plasmids were purified using the protocol Preparation of plasmid DNA by alkaline lysis with SDS: Minipreparation [67].

### 3.3. Prepro-Peptide cDNA Amplification and Selection 

The inserts linked in the plasmids were amplified using the forward and reverse M13 primers (Invitrogen), which annealed to the M13 reverse and forward priming sites present in the plasmid pCR^TM^4-TOPO^®^ by PCR according to the parameters specified: one cycle of 94 °C/120 s; 32 cycles of 94 °C/30 s, 53 °C/30 s, and 72 °C/60 s; and one cycle of 72 °C/420 s. Size evaluation of the insert fragments was performed by comparing band sizes in 2% agarose gels, (1X TAE buffer) and fragments with a molecular weight greater than 500 bp were selected for purification with the DNA PuriPrep-GP Kit (Highway). The quality of the amplified fragments was tested with agarose gels. 

### 3.4. Insert Sequencing and Prepro-Peptide Identification

Selected inserts were sequenced by the BigDye terminator reaction (Applied Biosystems), using an ABI 3130 DNA analyzer (Applied Biosystems) and universal M13 forward and reverse primers. Sequences were studied using the Lasergene sequence analysis software (DNASTAR, Inc., Madison, WI, USA). EMBOOS Transseq was used to translate nucleic acid sequences to the corresponding peptide sequences.

### 3.5. Sequence Analysis

The deduced peptide sequences were compared with those of the peptides deposited in the Antimicrobial Peptide Database (APD3) [12], Database of Antimicrobial Activity and Structure of Peptides (DBAASP) [68], DRAMP [21], and BLAST [69]. Several software packages were used for the characterization of the physical-chemical properties and the prediction of the activity of the sequences. The PEP-FOLD program [70] was used to predict the 3D structure of the peptide, UCSF Chimera was used for the renderings [71], and the HeliQuest program was used to evaluate amphipathic character [72]. The computer software DBAASP v2.702 [68] and the software ProtParam [73] were used to determine the physical and chemical parameters of the peptide sequences. The ClustalX tool of the MEGA7 software [74] was used to align the primary sequences of the peptides described for the genus *Leptodactylus*. The online program WebLogo [75] was used for the graphical representation of amino acid sequence alignment. The software packages DBAASP v2.702 and Collection of Anti-Microbial Peptides (CAMPR3) [76] allowed the prediction of antimicrobial activity, and the bioinformatics program Hemolytic Peptide Identification Server (HemoPI) [77] the prediction of hemolytic character.

## 4. Conclusions

The ocellatin peptides characterized to date belong exclusively to leptodactylid frogs. A review of the members of the ocellatin family shows that they have a length of 21, 25, or 32 amino acids, are cationic with net charges between +1 and +3, present a hydrophobic residue content between 42% and 57%, have a variable combination of physical-chemical properties, and have a high helix content, as indicated by 3D structure predictions.

The amino acid frequency analysis and particular motif occurrence demonstrates that certain amino acid residues in ocellatins are highly conserved or have conservative substitutions. Polar charged residues have a significant role in the electrostatic attraction between charged peptides and negatively charged bacterial membranes. SAR studies with ocellatins proved the effect of replacing charged residues by the electrically neutral Ala. The antibacterial activity of ocellatin-F proved to be dependent on the overall charge. The substitution of Asp_4_ or Asp_12_ for Ala resulted in increased antibacterial activity and the replacement of Lys with Ala resulted in decreased activity. Therefore, higher positively charged peptides will show greater capacity to disturb microorganism membranes. However, interestingly, it would seem that, for reasons unknown, ocellatins maintain a low cationic character as they preserve both the cationic amino acids and also the number of acidic residues in specific positions.

The present study was designed to identify novel peptides from the skin of the Argentinian frog *L. latrans*. Using molecular biology techniques to identify the complete prepro-peptide precursors, here, we describe five new members of the ocellatin family, namely ocellatin-7 to -11. The methodology used allowed us to obtain the entire sequence of mature peptide, which is relevant because ocellatin fragments were found to be always less active than ocellatins. This is the case of ocellatin-F (1–22) and ocellatin-L1 (1–22) fragments, which did not inhibit *E. coli*, while the mature peptides inhibited the strain at a concentration of 40 and 50 µM, respectively. Therefore, concerning the bioprospection of new molecules as antimicrobial alternatives, the identification of the entire prepro-peptide may be paramount.

The primary structure of the mature peptides identified indicates that they are orthologs of active peptides of the ocellatin family previously isolated from other species of South American frogs of the genus *Leptodactylus*. Ocellatin-7, -8, and -9 were found to be the members of the family with the lowest percentage of hydrophobic residues but with the highest amphipathicity µH values. Through comparison with peptides from the same family, we estimate that ocellatin-10 will be the most active of the five novel peptides, followed by ocellatin-7, -8, -9, and -11. 

These results increase the data available on the ocellatin peptide family. Future work will address the synthesis, purification, and testing of the activity of the new peptides described herein to corroborate the predictions. Further work with species of the *Leptodactylus* genus will be required to contribute to phylogeny relationship studies among species of this particular genus. Finally, we envisage that a general ocellatin structure could be used as a template for the preparation of libraries of peptides with improved antimicrobial activities. 

## Figures and Tables

**Table 1:** Theoretical molecular weight (Mw), total net charge, identity, and similarity percentage of newly identified ocellatin peptides.

Peptide	Sequence																						Mw	Net	Identity/Similarity	Reference
																								Charge	Percentage (%)	
ocellatin-2	G	V	L	D	I	F	K	D	A	A	K	Q	I	L	A	H	A	A	E	Q	I	^a^	2250.63	0		[15]
**ocellatin-11**	G	V	L	D	I	F	K	D	A	A	K	Q	I	L	A	H	A	A	E	K	I	^a^	2250.67	1	95.2/100	this work
ocellatin-5	A	V	L	D	I	L	K	D	V	G	K	G	L	L	S	H	F	M	E	K	V	^a^	2311.82	1		[27]
**ocellatin-7**	G	V	V	D	I	L	K	D	T	G	K	K	L	L	S	H	L	M	E	K	I	^a^	2336.87	2	71.4/76.2	this work
**ocellatin-8**	G	V	V	D	I	L	K	D	T	G	K	K	L	L	S	H	L	M	E	K	V	^a^	2322.84	2	76.2/76.2	this work
**ocellatin-9**	G	V	L	D	I	F	K	D	T	G	K	K	L	L	S	H	L	M	E	K	V	^a^	2370.89	1	57.1/76.2	this work
P3-Lla-2085	G	L	L	D	F	L	K	A	A	G	K	G	L	V	S	N	L	L	E	K	^a^		2085.52	2		[26]
**ocellatin-10**	G	L	L	D	F	L	K	A	A	G	K	G	L	V	S	N	L	I	E	K	^a^		2184.65	2	90.5/95.2	this work
ocellatin-6	A	I	L	D	F	I	K	A	A	G	K	G	L	V	T	N	I	M	E	K	V	G	2273.77	2	68.2/86.4	[27]

Boxed black letters represent identical amino acid position and boxed gray letters represent conservative substitutions. a: C-terminus amidated peptide. Net charge at pH 7.

**Table 2:** Amino acid sequence alignment of the 28 ocellatin peptides identified in the skin of *Leptodactylus* frog species.

Name		Sequence																														
Ocellatin-F (fallaxin)	G	V	V	D	I	L	K	G	A	A	K	D	I	A	G	H	L	A	S	K	V	M	N	K	L							
Ocellatin-K1	G	V	V	D	I	L	K	G	A	A	K	D	L	A	G	H	L	A	S	K	V	M	N	K	I							
Ocellatin-L1 (laticeptin)	G	V	V	D	I	L	K	G	A	A	K	D	L	A	G	H	L	A	T	K	V	M	N	K	L							
Ocellatin-L2	G	V	V	D	I	L	K	G	A	A	K	D	L	A	G	H	L	A	T	K	V	M	D	K	L							
Ocellatin-S (syphaxin)	G	V	L	D	I	L	K	G	A	A	K	D	L	A	G	H	V	A	T	K	V	I	N	K	I							
Ocellatin-P (pentadactylin)	G	L	L	D	T	L	K	G	A	A	K	N	V	V	G	S	L	A	S	K	V	M	E	K	L							
Ocellatin-7	G	V	V	D	I	L	K	D	T	G	K	K	L	L	S	H	L	M	E	K	I											
Ocellatin-8	G	V	V	D	I	L	K	D	T	G	K	K	L	L	S	H	L	M	E	K	V											
Ocellatin-9	G	V	L	D	I	F	K	D	T	G	K	K	L	L	S	H	L	M	E	K	V											
Ocellatin-5	A	V	L	D	I	L	K	D	V	G	K	G	L	L	S	H	F	M	E	K	V											
Ocellatin-10	G	L	L	D	F	L	K	A	A	G	K	G	L	V	S	N	L	I	E	K	V											
Ocellatin-6	A	V	L	D	F	I	K	A	A	G	K	G	L	V	T	N	I	M	E	K	V	G										
Ocellatin-PT6	G	V	F	D	I	I	K	G	A	G	K	Q	L	I	A	H	A	M	E	K	I	A	E	K	V	G	L	N	K	D	G	N
Ocellatin-PT8	G	V	F	D	I	I	K	G	A	G	K	Q	L	I	A	R	A	M	G	K	I	A	E	K	V	G	L	N	K	D	G	N
Ocellatin-PT7	G	V	F	D	I	I	K	G	A	G	K	Q	L	I	A	H	A	M	G	K	I	A	E	K	V	G	L	N	K	D	G	N
Ocellatin-PT4	G	V	F	D	I	I	K	G	A	G	K	Q	L	I	A	H	A	M	G	K	I	A	E	K	V							
Ocellatin-PT1	G	V	F	D	I	I	K	D	A	G	K	Q	L	V	A	H	A	M	G	K	I	A	E	K	V							
Ocellatin-PT5	G	V	F	D	I	I	K	D	A	G	R	Q	L	V	A	H	A	M	G	K	I	A	E	K	V							
Ocellatin-PT2	G	V	F	D	I	I	K	D	A	G	K	Q	L	V	A	H	A	T	G	K	I	A	E	K	V							
Ocellatin-PT3	G	V	I	D	I	I	K	G	A	G	K	D	L	I	A	H	A	I	G	K	L	A	E	K	V							
Ocellatin-V1	G	V	V	D	I	L	K	G	A	G	K	D	L	L	A	H	A	L	S	K	L	S	E	K	V							
Ocellatin-V3	G	V	L	D	I	L	T	G	A	G	K	D	L	L	A	H	A	L	S	K	L	S	E	K	V							
Ocellatin-V2	G	V	L	D	I	L	K	G	A	G	K	D	L	L	A	H	A	L	S	K	I	S	E	K	V							
Ocellatin-1	G	V	V	D	I	L	K	G	A	G	K	D	L	L	A	H	L	V	G	K	I	S	E	K	V							
Ocellatin-11	G	V	L	D	I	F	K	D	A	A	K	Q	I	L	A	H	A	A	E	K	I											
Ocellatin-2	G	V	L	D	I	F	K	D	A	A	K	Q	I	L	A	H	A	A	E	Q	I											
Ocellatin-4	G	L	L	D	F	V	T	G	V	G	K	D	I	F	A	Q	L	I	K	Q	I											
Ocellatin-3	G	V	L	D	I	L	K	N	A	A	K	N	I	L	A	H	A	A	E	Q	I											
	∙	:	∙	*		∙	∙		∙	∙	:		:				:			:	:											

Novel ocellatins identified in this work. Asterisks, colon, and dots indicates coincidences, and highly and weak conserved residues, respectively.

**Table 3:** Experimental results of antimicrobial activities of ocellatin family peptides.

Name	MIC				Reference
	*E. coli*		*S. aureus*		
	µg/mL	µM	µg/mL	µM	
Ocellatin-1	*	*	NT	NT	[15]
Ocellatin-2	*	*	NT	NT	[15]
Ocellatin-3	*	*	NT	NT	[15]
Ocellatin-4	145	64	145	64	[16]
Ocellatin-5	64	28	128	56	[27]
Ocellatin-6	32	14	64	28	[27]
Ocellatin-F (fallaxin)	106	40	>60	>160	[28] **
Ocellatin-F1	1060	397	293	110	[34] **
Ocellatin-P (pentadactylin)	64	25	508	200	[29]
Ocellatin-PT1	800	300	>800	>300	[18]
Ocellatin-PT2	>800	>310	>800	>310	[18]
Ocellatin-PT3	800	320	>800	>320	[18]
Ocellatin-PT4	200	80	>800	>310	[18]
Ocellatin-PT5	800	300	>800	>300	[18]
Ocellatin-PT6	400	120	>800	>240	[18]
Ocellatin-PT7	200	60	800	245	[18]
Ocellatin-PT8	200	60	800	245	[18]
Ocellatin-L1 (laticeptin)	128	50	512	>200	[30]
Ocellatin-L2	I	I	I	I	[30]
Ocellatin-S (syphaxin)	NT	NT	I	I	[31]
Ocellatin-V1	512	>200	512	>200	[32]
Ocellatin-V2	514	>200	514	>200	[32]
Ocellatin-V3	I	I	I	I	[32]
Ocellatin-K1	NI	NI	NI	NI	[33]
P3-Lla-2085	31	15	31	15	[26]

* Evaluation of inhibition zones using an in vitro assay on LB-agarose plates using 2–4 µg/µL. ** Difference of an order of magnitude between the two authors. NT: not tested. NI: not informed. I: inactive.

## Supplementary Materials

The following are available online at https://www.mdpi.com/2079-6382/9/11/751/s1. Figure S1. Size evaluation of the amplified insert fragments in 2% agarose gels.; Figure S2. Fragments selected for sequencing.; Figure S3. Nucleic acid and deduced amino acid sequence of cDNA encoded ocellatin-7, ocellatin-8, ocellatin-9, ocellatin-10, ocellatin-11 from the skin of *Leptodactylus latrans*.; Figure S4. Amino acid sequence alignment of the five novel peptides identified in the skin of *Leptodactylus latrans*; Figure S5. Hydrophobic moment (µH), Hydrophobicity (GRAVY), percentage of α-helix, and percentage of aggregation of the ocellatins described to date; Figure S6. Ocellatins with α-helix 3D theoretical structure; Figure S7. Ocellatins with 3D theoretical structure of two α-helix linked by a kink; Figure S8. Schiffer and Edmundson wheel projection diagrams of ocellatins with complete α-helix 3D structure prediction.; Table S1: Ocellatins and other peptides described to date from the skin secretion of Leptodactylid frogs. In vivo and in silico parameters; Table S2. Similarity percentages of the identified ocellatins by pairwise alignment; Table S3. Fragments of ocellatin peptides identified in the skin secretion of frog of the *Leptodactylus* genus; Supporting information: 3D structures.
