# Supplementary material for: Identification of New Ocellatin Antimicrobial Peptides by cDNA Precursor Cloning in the Frame of This Family of Intriguing Peptides
Source: Antibiotics (Basel). 2020 Oct 29;9(11):751. doi: 10.3390/antibiotics9110751 (PMC7693824; doi:10.3390/antibiotics9110751)
Supplement: Supplementary file 1 [file antibiotics-09-00751-s001.pdf]

Supporting Information

Identification of New Ocellatin Antimicrobial Peptides by cDNA Precursors Cloning in the Frame of this Family of Intriguing Peptides

Mariela M. Marani[1*], Silvana Aguilar[1], Ana P. Cuzziol Boccioni[1,2], Natalia L. Cancelarich[1], Néstor G. Basso[3], Fernando Albericio[4,5]

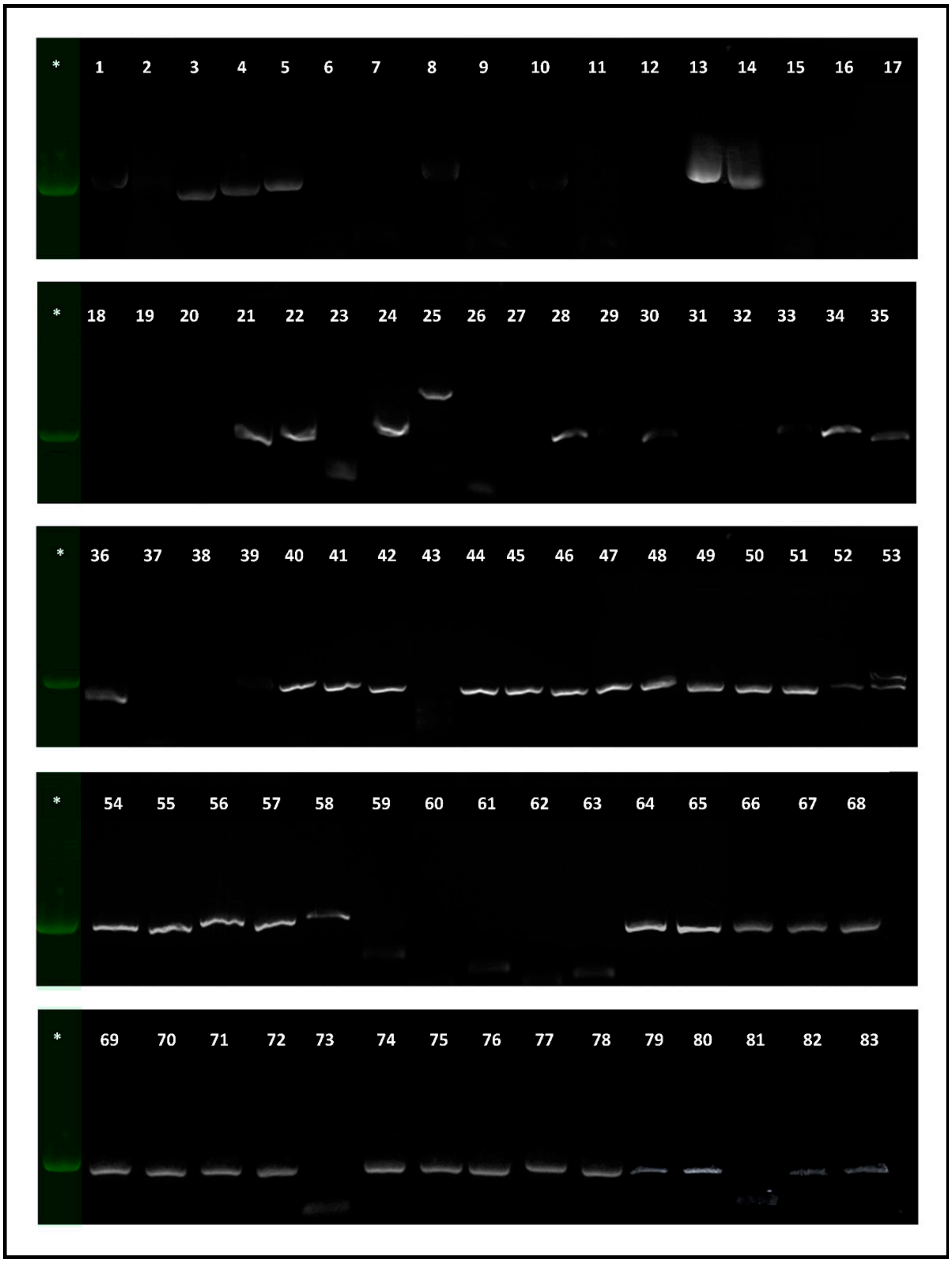

Figure S1. *Size evaluation of the amplified insert fragments in 2% agarose gels. The inserts linked in the plasmids pCR™4-TOPO® were amplified using the forward and reverse M13 primers. The asterisk (*) indicates to a reference fragment of 487 base pairs.*

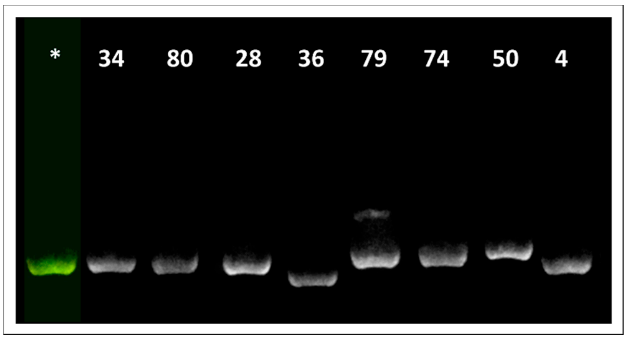

Figure S2. *Fragments selected for sequencing. The asterisk (*) indicates to a reference fragment of 487 base pairs.*

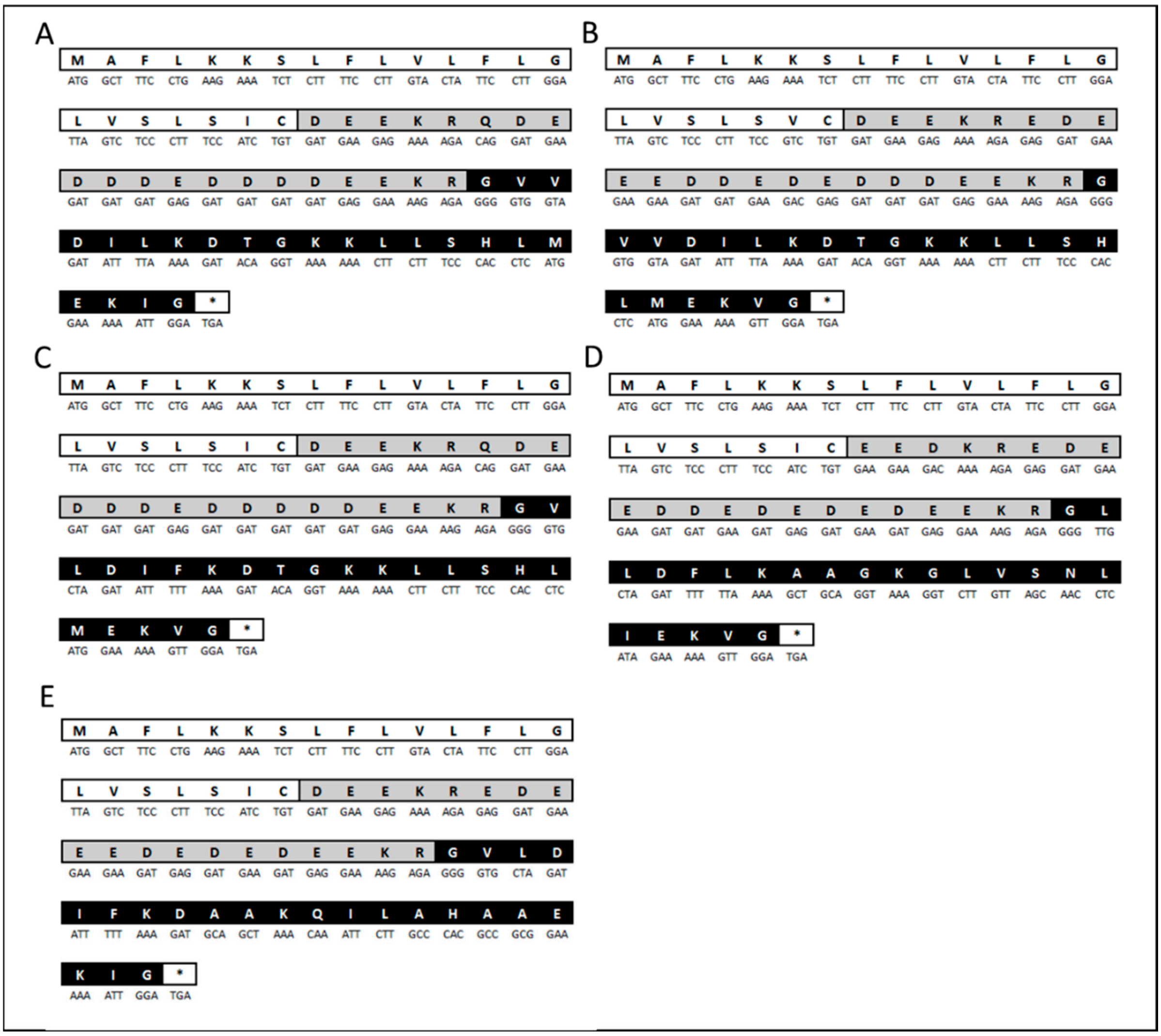

Figure S3. *Nucleic acid and deduced amino acid sequence of cDNA encoded A) ocellatin-7, B) ocellatin-8, C) ocellatin-9, D) ocellatin-10, ocellatin-11 from the skin of Leptodactylus latrans. Signal peptide, acidic region, and mature peptide are signaled in white, gray, and black boxed-letters respectively, and stop codon with an asterisk.*

```
CLUSTAL multiple sequence alignment by MUSCLE (3.8)


Ocellatin-11      GVLDIFKDAAKQILAHAAEKI
Ocellatin-7       GVVDILKDTGKKLLSHLMEKI
Ocellatin-8       GVVDILKDTGKKLLSHLMEKV
Ocellatin-9       GVLDIFKDTGKKLLSHLMEKV
Ocellatin-10      GLLDFLKAAGKGLVSNLIEKV
                  *::*::* :.* ::::  **:
```

Figure S4. *Amino acid sequence alignment of the five novel peptides identified in the skin of Leptodactylus latrans.*

Table S2. Similarity percentages of the identified ocellatins by pairwise alignment.

	SIMILARITY (%)				
	**Ocellatin-7**	**Ocellatin-8**	**Ocellatin-9**	**Ocellatin-10**	**Ocellatin-11**
**Ocellatin-7**		100.0	90.5	61.9	71.4
**Ocellatin-8**	100.0		90.5	61.9	71.4
**Ocellatin-9**	90.5	90.5		61.9	81.0
**Ocellatin-10**	61.9	61.9	61.9		52.4
**Ocellatin-11**	71.4	71.4	81.0	52.4	

Table S3. Fragments of ocellatin peptides identified in the skin secretion of frog of the *Leptodactylus* genus.

Species	Original fragment name/proposed name	Sequence	MIC (μM) *E. coli*	Reference
L. fallax	ocellatin-F (1-22)	GVVDILKGAAKDIAGHLASKVM	ND	[1]
L. laticeps	ocellatin-L1 (1-22)	GVVDILKGAAKDLAGHLATKVM	NT	[2]
L. syphax	syphaxin (1-16)/ocellatin-S (1-16)	GVLDILKGAAKDLAGH	10,1	[3]
	syphaxin (1-19)/ocellatin-S (1-19)	GVLDILKGAAKDLAGHVAT	NT	
	syphaxin (1-22)/ocellatin-S (1-22)	GVLDILKGAAKDLAGHVATKVI	3,6	
	syphaxin (1-23)/ocellatin-S (1-23)	GVLDILKGAAKDLAGHVATKVIN	NT	
	syphaxin (1-24)/ocellatin-S (1-24)	GVLDILKGAAKDLAGHVATKVINK	NT	
	syphaxin (16-25)/ocellatin-S (16-25)	HVATKVINKI	NT	
L. latrans	ocellatin-1.1/ocellatin-1 (1-16)	GVVDILKGAGKDLLAH	NT	[4]
	ocellatin-2.1/ocellatin-2 (1-15)	GVLDIFKDAAKQILA	NT	
	ocellatin-3.1/ocellatin-3 (1-15)	GVLDILKNAAKNILA	NT	
	ocellatin-5.1/ocellatin-5 (1-14)	AVLDILKDVGKGLL	NT	
	ocellatin-6.1/ocellatin-6 (1-18)	AVLDFIKAAGKGLVTNIM	NT	
	P2-L1-1298*	AAGKGLVSNLLEK	24,6	[5]
	ocellatin-5*	GLLDFLKAAGKGLVTNL	**	[6]
L .labyrinticus	ocellatin-LB1/ocellatin-F (1-22)	GVVDILKGAAKDIAGHLASKVM	114	[7]
	ocellatin-LB2; Des-Lys^{24}-Leu^{25}-OF1§/ocellatin-F (1-23)	GVVDILKGAAKDIAGHLASKVMN	ND	[7; 8]
L. vastus	ocellatin-K1 (1-16)***	GVVDILKGAAKDLAGH	125	[9]
	ocellatin-K1 (1-21)***	GVVDILKGAAKDLAGHLASKV	125	

ND= not detected

NT= not tested

* the complete sequence of the derivative ocellatin has not already been described.

** not accessible information

***also mitigate LPS-induced ROS formation and NF-kB activation in microglia and hippocampal neurons

§ mild antiviral effect on the inhibition of rabies virus infection

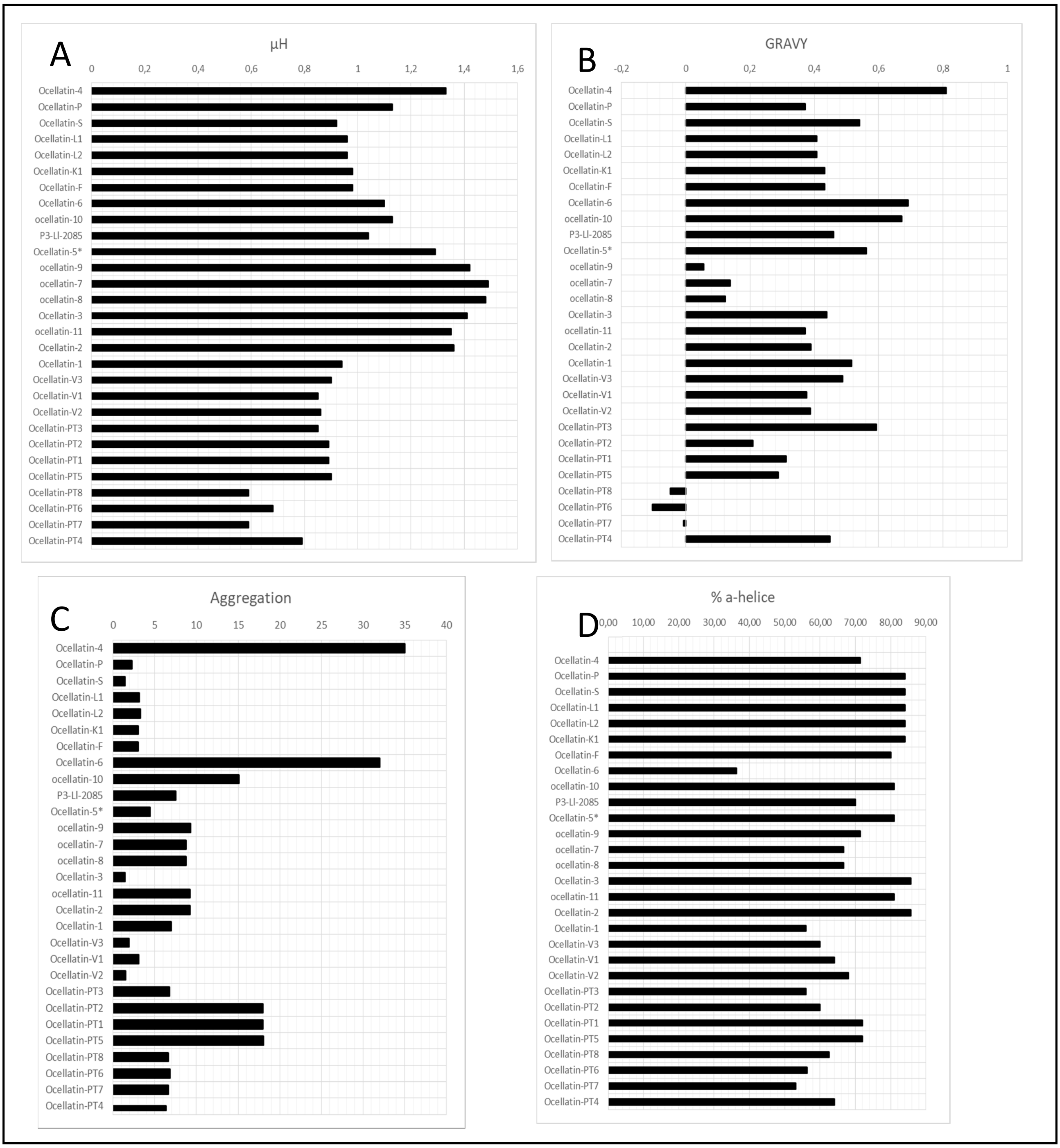

Figure S5. *A) Hydrophobic moment (μH), B) Hydrophobicity (GRAVY), C) percentage of aggregation and D) percentage of α-helix of the ocellatins described to date.*

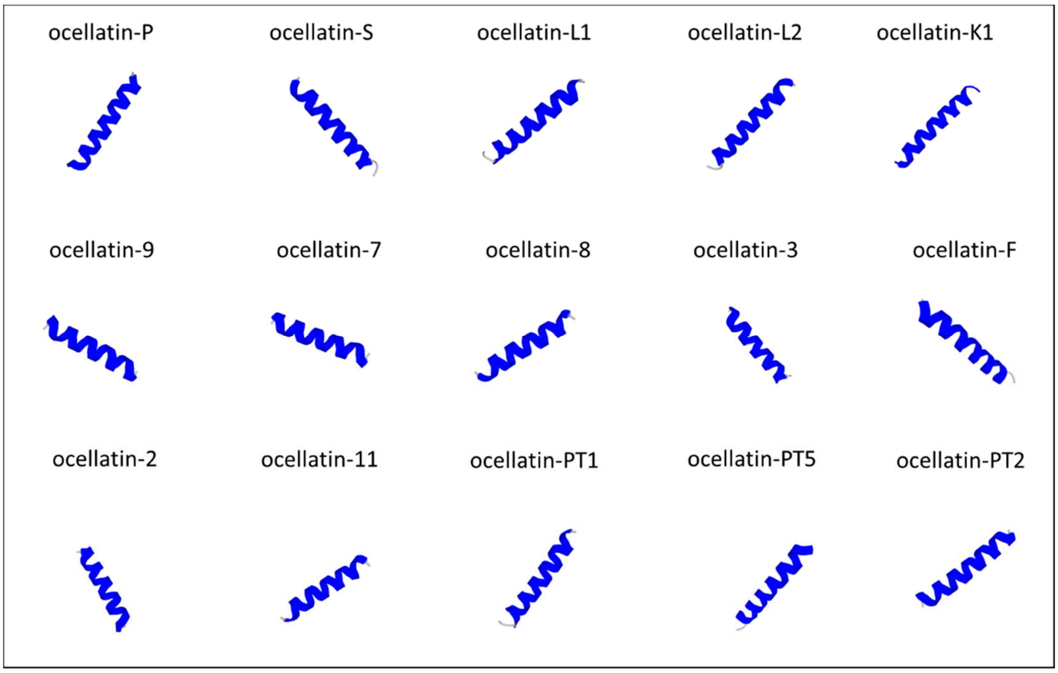

Figure S6. *Ocellatins with α-helix 3D theoretical structure.*

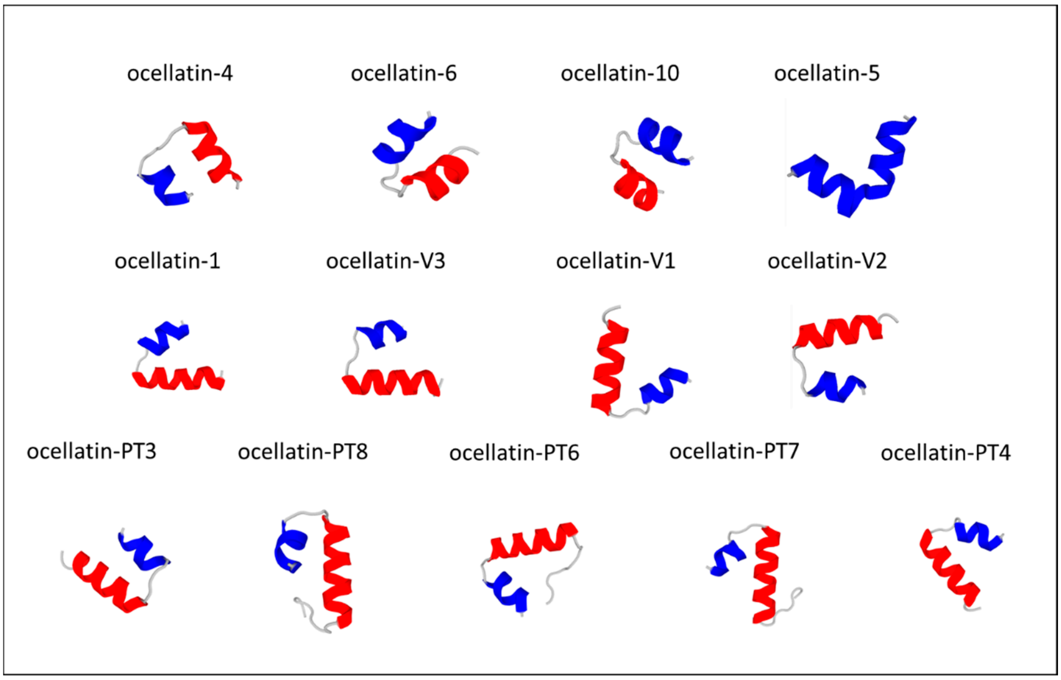

Figure S7. *Ocellatins with 3D theoretical structure of two α-helix linked by a kink.*

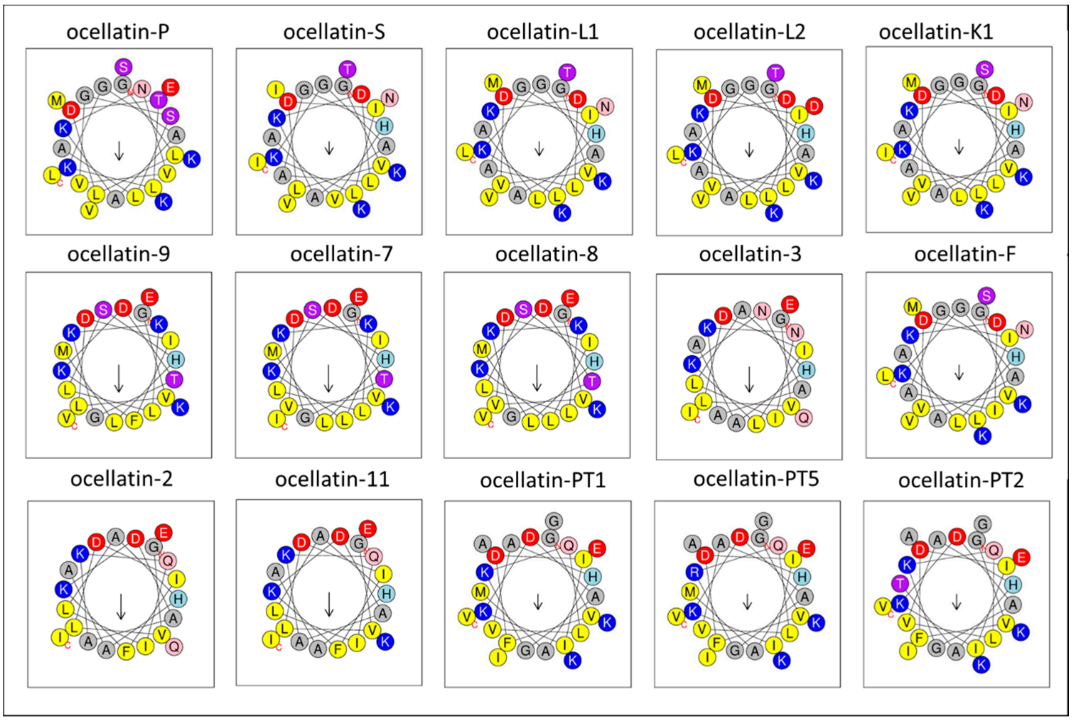

Figure S8. *Schiffer and Edmundson wheel projection diagrams of ocellatins with complete α-helix 3D structure prediction. The size of the vector indicates the amphipaticity of the helix.*

3D structures

In general, the presence of particular amino acids such as Gly and Pro caused twists, but it depends on the environment in which they are located [10] (Xia & Xie, 2002). For the entire ocellatin family, the X10 position varies between only two amino acids Ala and Gly, followed by a Lys that is invariant for all ocellatins. When Ala is in site 10, the peptide adopts an entire α-helix structure, while when Gly10 is present, the helix is twisted independently of the flanking amino acids. It is valid for all the AMPs with AAKQ, AAKD, AAKN (whole helix) and AGKG, AGKD, AGKQ, VGKG, VGKD (twisted helix) motifs, not only in ocellatin family peptides but also with antimicrobial peptides from other isolated amphibian families. The motif AGKG producing a twisted-helix it is also present in brevinin-2GHc and several peptides of the nigrocin and nigroanin family (nigrocin-2Isa, -2ISb, -2ISc, and nigroanin-K2, -K-SN1). Besides, the motif AGKQ is present in palustrin-2LTa, and nigrocin-OG5, and the motif VGKG in dermatotoxin A1. Whereas the motif AAKN, and therefore a helical structure in that region, is observed in six peptides of the ranatuerin family (ranatuerin-2Ple, -2PRa,-2Ara,-2CPc, -2CHa, and -2PLx), the motif AAKD in five ranatuerin peptides (ranatuerin-2, -2Ca, -2PLb, -2PLf, -2G) and palustrin-2DY1, and the motif AAKQ even in peptides isolated from other organisms as polybia-MPI from arthropods and two cupienins from spiders (supporting Information). Interestingly, 3 of the new ocellatins present the motif TGKK and the 3D theoretical structure is an exception for the above-cited. This motif (TGKK with Gly at position 10) is not present in neither of 1101 identified antimicrobial peptides from amphibians to date. Only is present in 4 of the 3217

peptides of the Antimicrobial Peptide Database, two isolated from bacteria (warnerin y epilancin), one from human (2L4N), and one from porcine (PorcineNK) but not in the studied position. Also is observed one exception in the ocellatin-PT1, PT-2 y PT-5. These exceptions could be due to the presence of Asp in site 8. The existence of the motif $D_8XG_{10}K_{11}$, were X is any of the 20 amino acids, is very unusual in antimicrobial peptide sequences described to date. Some examples are palustrin-Ca present in the skin of the frog *Lithobates catesbeianus* (motif $D_8T_9G_{10}K_{11}$) [11] (**Zhao et al., 2011**) and Brevinin-2RTa of *Amolops ricketti* (motif $D_8F_9G_{10}K_{11}$) [12] (**Wang et al., 2011**) wherein both molecules a unique helix is predicted (without a twist although it present $G_{10}K_{11}$).

References

[1]Rollins-Smith, L.A.; King, J.D.; Nielsen, P.F.; Sonnevend, A.; Conlon, J.M. An antimicrobial peptide from the skin secretions of the mountain chicken frog Leptodactylus fallax (Anura:Leptodactylidae). *Regul. Pept.* **2005**, *124*, 173–178.

[2]Conlon, J.M.; Al-Ghaferi, N.; Abraham, B.; Sonnevend, A.; King, J.D.; Nielsen, P.F. Purification and properties of laticeptin, an antimicrobial peptide from skin secretions of the South American frog *Leptodactylus laticeps*. *Protein Pept. Lett.* **2006**, *13*, 411–415.

[3]Dourado, F.S.; Leite, J.R.S.A.; Silvab, L.P.; Melo, J.A.T.; Bloch, C. Jr.; Schwartz, E.F. Antimicrobial peptide from the skin secretion of the frog *Leptodactylus syphax. Toxicon* **2007**, *50*, 572–580.

[4]Leite, J.M.A., Jr.; Silva, L.P.; Silva-Leite, R.R.; Ferrari, A.S.; Noronha, S.E.; Silva, H.R.; Bloch, C., Jr.; Leite, J.R.S.A. *Leptodactylus Ocellatus* (Amphibia): mechanism of defense in the skin and molecular phylogenetic relationships. *J. Exp. Zool.* **2010**, *313* A, 1–8.

[5] Siano, A.; Humpola, M.V.; de Oliveira, E.; Albericio, F.; Simonetta, A.C.; Lajmanovich, R.; Tonarelli, G.G. *Leptodactylus latrans* Amphibian Skin Secretions as a Novel Source for the Isolation of Antibacterial Peptides. *Molecules* **2018**, *23*, 2943.

[6]Nascimento A.C.C. Cytolytic peptides and proteases from the skin secretion of the frog *Leptodactylus ocellatus*. University of Brasilia, Brazil. 2007. https://www.uniprot.org/uniprot/P85443

[7]Gusmão, K.A.G.; dos Santos, D.M.; Santos, V.M. et al. Ocellatin peptides from the skin secretion of the South American frog *Leptodactylus labyrinthicus* (Leptodactylidae): characterization, antimicrobial activities and membrane interactions. *J Venom Anim Toxins Incl Trop Dis* **2017**, *23*, 4.

[8]Cunha Neto, R.D.; Vigerelli,H.; Jared, C.; Antoniazzi, M.M.; Chaves, L.B.; Rodrigues da Silva, A.C.; Lopes de Melo, R.; Sciani, J.M.; Pimenta, D.C. Synergic effects between ocellatin-F1 and bufotenine on the inhibition of BHK-21cellular infection by the rabies virus. *J. Venom. Anim. Toxins* **2015**, *21*(50).

[9]Sousa, N.A.; Oliveira, G.A.L.; de Oliveira, A.P. et al. Novel Ocellatin Peptides Mitigate LPS-induced ROS Formation and NF-kB Activation in Microglia and Hippocampal Neurons. *Sci Rep* **2020**, 10, 2696.

[10]Xuhua X., Zheng X. Protein Structure, Neighbor Effect, and a New Index of Amino Acid Dissimilarities, *Molecular Biology and Evolution,* **2002,** *19* (1) 58–67, https://doi.org/10.1093/oxfordjournals.molbev.a003982

[11]Zhao R.L., Han J.Y., Han W.Y., He H.X., Ma J.F. Effects of two novel peptides from skin of *Lithobates catesbeianus* on tumor cell morphology and proliferation. **2011.** *Molecular Cloning – Selected Applications in Medicine and Biology.* Chapter 5.

[12]Wang H, Ran R, Yu H, Yu Z, Hu Y, Zheng H, Wang D, Yang F, Liu R, Liu J. Identification and characterization of antimicrobial peptides from skin of Amolops ricketti (Anura: Ranidae). *Peptides.* **2012,** *33* (1) 27–34.
